# Circulating levels of soluble Dipeptidylpeptidase-4 are reduced in human subjects hospitalized for severe COVID-19 infections

**DOI:** 10.1038/s41366-020-00689-y

**Published:** 2020-09-21

**Authors:** Kristina Schlicht, Nathalie Rohmann, Corinna Geisler, Tim Hollstein, Carina Knappe, Katharina Hartmann, Jeanette Schwarz, Florian Tran, Domagoj Schunk, Ralf Junker, Thomas Bahmer, Philip Rosenstiel, Dominik Schulte, Kathrin Türk, Andre Franke, Stefan Schreiber, Matthias Laudes

**Affiliations:** 1 Division of Endocrinology, Diabetes and Clinical Nutrition, Department of Medicine 1, University of Kiel, Kiel, Germany; 2 Institute of Clinical Chemistry, University of Kiel, Kiel, Germany; 3 Institute of Clinical Molecular Biology, University of Kiel, Kiel, Germany; 4 Division of Pneumology, Department of Medicine 1, University of Kiel, Kiel, Germany; 5 Interdisciplinary Emergency Center, University of Kiel, Kiel, Germany

**Keywords:** Obesity, Risk factors, Type 2 diabetes, Enzymes

## Abstract

Dipeptidylpeptidase (DPP)-4 is a key regulator of the incretin system. For several years DPP-4 inhibitors in addition to GLP-1 analogues are of major importance in the clinical management of obesity and type 2 diabetes. DPP-4 is also known as CD26 and represents a membrane bound protease on the surface of several eukaryotic cell types. Of interest, DPP-4, like ACE2, has been shown to serve as a binding partner for corona-like viruses to enter host immune cells. Since metabolic diseases are major risk factors for the present COVID-19 pandemic, we examined circulating soluble DPP-4 serum concentrations in patients suffering from severe COVID-19 infection and in healthy human subjects in a case control design. In this analysis sDPP-4 levels were significantly lower in COVID-19 patients compared to controls (242.70 ± 202.12 ng/mL versus 497.70 ± 188.13 ng/mL, p = 0.02). We also examined sDPP-4 serum concentrations in patients suffering from sepsis not due to corona-like viruses. In these subjects, sDPP-4 levels were not different compared to healthy case controls (p = 0.14), which might suggest the decrease of sDPP-4 to be specific for corona-like virus infections. Currently, most data point towards membrane bound ACE2 in contrast to DPP-4 as the major binding partner for COVID-19 internalization into host immune cells. However, the finding that the circulating soluble form of DPP-4 is reduced in hospitalized patients might suggest a regulatory role for both, ACE and DPP-4, in COVID-19 infections, especially since obesity and type 2 diabetes are major risk factor for a severe course of the disease

## Introduction

The recent outbreak of the corona virus COVID-19 in Wuhan, China, has spread globally resulting in an unprecedented threat of millions of human subjects all around the world [1]. Of interest, obesity and type 2 diabetes are stronger risk factors compared to chronic pulmonary diseases for a severe course of the infection with the need for intensive care treatment [2]. Indeed, cigarette smoking, known to cause pulmonary damage, might even be associated with milder forms of COVID-19 infections [3]. Beside evidence of metabolic diseases being risk factors for a severe disease course, observations from other corona-like virus outbreaks (SARS-CoV in 2002–2003 and MERS-CoV in 2012–2015) revealed long term metabolic complications even 12 years after acute infection in survivors [4]. Thus, molecular, cellular and clinical studies on the relation of COVID-19 and human metabolism might be important for both, to improve acute treatment of the infection and also to prevent long term metabolic complications of the millions of infected subjects worldwide. In the past it has been shown (1.) that membrane bound proteases like *Dipeptidylpeptidase* (DPP)-4 and *Angiotensin Converting Enzyme* (ACE)2 serve as binding partners for corona-like viruses to enter host immune cells [5] and (2.) that the circulating soluble form of DPP-4 (=sDPP-4) is reduced in MERS-CoV affected subjects [6]. Therefore, in the present study we examined circulating sDPP-4 serum concentrations in human COVID-19 patients compared to healthy controls in order to gain evidence, whether sDPP-4 could serve as a potential future target to improve acute and long-term COVID-19 metabolic comorbidities.

## Patients and methods

### Study design

A case control study design was used to assess whether circulating sDPP-4 serum concentrations in *n* = 7 patients suffering a severe course of COVID-19 were different from healthy controls. Therefore, healthy controls from the cross-sectional Food Chain Plus (FoCus) cohort were matched in a 1 case to 2 controls (1:2) ratio using recruiting age, sex and BMI. As part of this population-based cohort, extensive subject data has been collected from 2011 to 2014, including phenotypic parameters and serum blood samples. As a comparison, sDPP-4 serum concentrations of *n* = 8 patients suffering from sepsis independent of COVID-19 infection were also evaluated. For this purpose, these samples were also matched with FoCus controls in a 1:2 ratio using age, sex and BMI. Exclusion criterion for healthy controls was the diagnosis of any chronic inflammatory disease, e.g. Inflammatory Bowel Disease or Rheumatoid arthritis.

### Characterization of the study cohort

The average age of the COVID-19 patients was 66.29 ± 11.70 and mean BMI was 24.26 ± 2.31. In healthy controls, the mean age was 62.43 ± 9.43 and mean BMI was 24.22 ± 4.04. One of the 7 COVID-19 patients was female, while the healthy control group consisted of male participants only. In the sepsis group, consisting of 4 male and 4 female patients, mean age was 65.00 ± 14.57 and mean BMI was 33.13 ± 12.10.

### sDPP-4 measurement

sDPP-4 serum concentration was measured using the Enzyme-linked immunosorbent assay (ELISA)-Kit from Abcam (Abcam, Cambridge, MA, USA, ab22287) according to the manufacturer’s instructions.

### Statistical analysis

Statistical analysis was carried out with R (R Core Team, 2020) and RStudio Version 1.2.5033 (RStudio., Inc., Boston, Massachusetts, USA). Continuous variables were tested for normal distribution with Shapiro–Wilk test. Since all data are normally distributed, they are presented as mean ± standard deviation. To evaluate differences between cases and controls, *t*-test for normally distributed data was used. Statistical level was set at alpha ≤ 0.05.

## Results and discussion

DPP-4, also known as CD26, not only exists in a membrane bound manner but also in a soluble form (= sDPP-4) which circulates systemically [7]. Plasma sDPP-4 activity was found to be altered in obesity and to be related to several components of the metabolic syndrome [8]. Previous studies have proposed that sDPP-4 is released from adipose tissue as a pro-inflammatory adipokine linking insulin resistance to low-grade inflammation [9–11]. Of interest, for the MERS-CoV corona-like virus it was explicitly shown that CD26 is the important membrane bound protein mediating internalization of the virus into host immune cells. Treatment with sDPP-4 has been proven to block the entry of corona like viruses into host cells via competitive inhibition in vitro and lower serum levels of sDPP-4 have been found in MERS-CoV infected human subjects compared to healthy controls in vivo [6].

Using a modelling approach, a recent publication demonstrated that the S1 domain of the Sars-CoV-2 spike glycoprotein might interact with membrane bound human DPP-4 [12]. In contrast, functional assays suggest ACE2 to be the major binding partner for COVID-19 and DPP-4 to be important for MERS-CoV corona-like virus internalization into host cells [13]. However, from our point of view the fact that COVID-19 internalizes predominantly via ACE2 does not rule out additional binding of the soluble form of DPP-4 in the systemic circulation, especially since obesity and type 2 diabetes are major risk factors for a severe course of a COVID-19 disease.

**Fig. 1:**
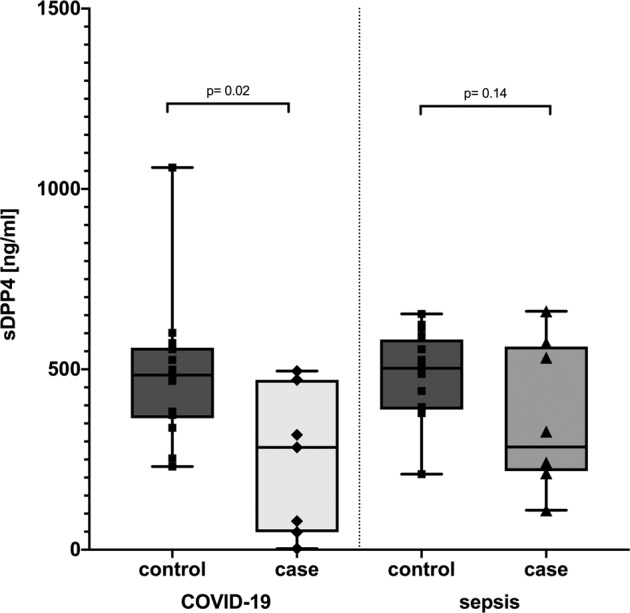
sDPP-4 in patients suffering from COVID-19 and matched healthy controls, as well as in sepsis patients and matched healthy controls. sDPP-4 is significantly reduced in patients suffering from acute COVID-19 infection compared to healthy controls (*p* *=* 0.02). sDPP4 is not significantly reduced in sepsis patients, compared to healthy controls (*p* *=* 0.14). Statistical significance was tested using *t-*Tests.

In the present study we determined serum concentrations of circulating sDPP-4 in *n* = 7 human subjects hospitalized for a severe COVID-19 disease course in the university hospital of Kiel in northern Germany. Five out of seven subjects recovered from the acute infection and have been discharged from the hospital. Two patients died in the course of the disease: The female patient being comorbid due to an exulcerated breast cancer, chronic obstructive pulmonary disease, heart insufficiency and coronary artery disease. A male patient died from multiple organ failure. Mean BMI accounted to 24.26 ± 2.31, two patients were overweight and one patient suffered from type 2 diabetes. Given the small sample size we used a case control design for the statistical analysis by recruiting age, sex and BMI matched controls from our cross-sectional FoCus cohort comprising almost 2000 human individuals [14]. Of interest, as it was found for MERS-CoV corona-like virus infections [6], we identified a significant decrease in circulating sDPP-4 in the serum of COVID-19 patients compared to controls (*p* = 0.02) [Fig. 1]. In order to examine if this finding represents an unspecific reaction during a severe acute infection, we also examined circulating sDPP-4 serum concentrations in human subjects suffering of sepsis not due to COVID-19. Of interest, in these sepsis patients, sDPP-4 concentrations were not significantly altered compared to case controls (*p* = 0.14) with a mean sDPP4 concentration of 361.27 ± 200.12 ng/ml, suggesting the sDPP-4 decrease to be a specific mechanism for the corona-like viruses MERS-CoV and COVID-19.

It is presently discussed that anti-diabetic medications of the class of the DPP-4 inhibitors might affect the individual disease course of COVID-19 [15] and a clinical trial with linagliptin as a potential COVID-19 treatment has been initiated [NCT04341935]. The results from the functional assays showing that ACE2 but not DPP-4 is the important membrane bound protein for virus internalization might question these hypotheses. However, several researchers (see [16] for a summary) have speculated about the role of DPP-4 as an important co-receptor of COVID-19. The fact (1) that the S1 domain of COVID-19 spike glycoprotein is able to interact with DPP-4, (2) that sDPP-4 is reduced in COVID-19 patients as in MERS-CoV infected subjects and (3) that obesity and type 2 diabetes are major risk factor for a severe course of COVID-19, from our point of view represent enough scientific evidence for further research into the potential role of circulating sDPP-4 in COVID-19 acute and long-term metabolic complications.

## Data Availability

Data and biomaterials are stored at the biobank P2N and can be requested there (https://portal.popgen.de).

## References

[1] Velavan T, Meyer C (2020). The COVID-19 epidemic. Trop Med Int Health.

[2] Zheng Z, Peng F, Xu B, Zhao J, Liu H, Peng J (2020). Risk factors of critical & mortal COVID-19 cases. J Infection.

[3] Miyara M, Tubach F, Pourcher V, Morelot-Panzini C, Pernet J, Haroche J, et al. Low incidence of daily active tobacco smoking in patients with symptomatic COVID-19. Qeios ID: WPP19W.3. 2020.

[4] Wu Q, Zhou L, Sun X, Yan Z, Hu C, Wu J (2017). Altered lipid metabolism in recovered SARS patients twelve years after infection. Sci Rep.

[5] Raj V, Mou H, Smits S, Dekkers D, Müller M, Dijkman R (2013). Dipeptidyl peptidase 4 is a functional receptor for the emerging human coronavirus-EMC. Nature.

[6] Inn K-S, Kim Y, Aigerim A, Park U, Hwang E-S, Choi M-S (2018). Reduction of soluble dipeptidyl peptidase 4 levels in plasma of patients infected with Middle East respiratory syndrome coronavirus. Virology.

[7] Lambeir A-M, Durinx C, Scharpé S, Meester I (2003). Dipeptidyl-peptidase IV from bench to bedside. Crit Rev Clin Lab Sci.

[8] Trzaskalski N, Fadzeyeva E, Mulvihill E (2020). Dipeptidyl peptidase-4 at the interface between inflammation and metabolism. Clin Med Insights Endocrinol Diabetes.

[9] Lamers D, Famulla S, Wronkowitz N, Hartwig S, Lehr S, Ouwens D (2011). Dipeptidyl peptidase 4 is a novel adipokine potentially linking obesity to the metabolic syndrome. Diabetes.

[10] Sell H, Blüher M, Klöting N, Schlich R, Willems M, Ruppe F (2013). Adipose dipeptidyl peptidase-4 and obesity. Diabetes care.

[11] Zilleáen P, Celner J, Kretschmann A, Pfeifer A, Racké K, Mayer P (2016). Metabolic role of dipeptidyl peptidase 4 (DPP4) in primary human (pre)adipocytes. Sci Rep.

[12] Vankadari N, Wilce J (2020). Emerging COVID-19 coronavirus. Emerg Microbes Inf.

[13] Hoffmann M, Kleine-Weber H, Schroeder S, Krüger N, Herrler T, Erichsen S (2020). SARS-CoV-2 cell entry depends on ACE2 and TMPRSS2 and Is blocked by a clinically proven protease inhibitor. Cell.

[14] Relling I, Akcay G, Fangmann D, Knappe C, Schulte D, Hartmann K (2018). Role of wnt5a in metabolic inflammation in humans. J Clin Endocrinol Metab.

[15] Iacobellis G (2020). COVID-19 and diabetes. Diabetes Res Clin Pract.

[16] Solerte S, Di Sabatino A, Galli M, Fiorina P (2020). Dipeptidyl peptidase-4 (DPP4) inhibition in COVID-19. Acta Diabetol.

